# Infusion parameters, safety, and practical guidance for the manual administration of subcutaneous immunoglobulin 20% (Ig20Gly)

**DOI:** 10.1186/s13223-024-00914-7

**Published:** 2024-10-04

**Authors:** Dorothea Grosse-Kreul, Crystal Allen, Chrystyna Kalicinsky, Paul K. Keith

**Affiliations:** 1 School of Immunology and Microbial Sciences, Department of Immunological Medicine and Allergy, King’s College Hospital NHS Foundation Trust, King’s Health Partners, London, UK; 2 Clinical Epidemiology Program, The Ottawa Hospital Research Institute, Ottawa, ON Canada; 3 Department of Nursing, The Ottawa Hospital, Ottawa, ON Canada; 4 Department of Infectious Diseases, Ontario Immunoglobulin Treatment (ONIT) Program, The Ottawa Hospital, Ottawa, ON Canada; 5 Department of Internal Medicine, Section of Allergy and Clinical Immunology, University of Manitoba, Winnipeg, MB Canada; 6 Division of Clinical Immunology and Allergy, Department of Medicine, McMaster University, Hamilton, ON Canada

**Keywords:** Home infusion, Inborn errors of immunity, Manual push, Primary immunodeficiencies, Rapid push, SCIG 20%, Treatment individualization

## Abstract

Primary immunodeficiency diseases (PIDs), also referred to as inborn errors of immunity, constitute a group of genetic conditions that affect the immune system. The current standard of care for patients with PIDs is lifelong immunoglobulin replacement therapy, delivered by intravenous (IVIG) or subcutaneous (SCIG) infusion. Immune globulin subcutaneous (human) 20% solution stabilized with glycine (Ig20Gly) is indicated as a replacement therapy for PIDs in adults and children of any age in Europe and in patients aged 2 years and above in the USA. Typically, Ig20Gly is administered using an infusion pump; however, delivery of Ig20Gly by manual administration has recently been approved in Europe. Practical recommendations on the use of Ig20Gly manual administration are lacking; this review therefore aims to provide guidance for use of this method of administration. Additionally, we summarize the infusion parameters, safety, patient-reported outcomes, and economic benefits associated with Ig20Gly manual administration. Manual administration of Ig20Gly was shown to permit faster rates of infusion than administration via infusion pump. Patients typically infused at two or fewer infusion sites with manual administration of Ig20Gly. Safety and tolerability profiles were similar for Ig20Gly manual administration and administration by infusion pump. Overall, there were comparable levels of patient satisfaction with manual administration and infusion pump, with patient preference deemed to be a key determinator of success for either method of administration. Economic studies identified cost savings for the healthcare system through manual administration compared with IVIG or SCIG infusion by infusion pump because of the reduced equipment costs and nurse support. For infusion of Ig20Gly by manual administration, a syringe and butterfly needle are used; patients are advised to start infusion at 1–2 mL/min to prevent discomfort. Overall, manual administration of Ig20Gly offers an effective and well-tolerated alternative to administration by infusion pump.

## Background

Primary immunodeficiency diseases (PIDs), also referred to as inborn errors of immunity, are a group of approximately 485 genetic conditions that affect the immune system [1, 2]. Clinical manifestations of PIDs are highly variable and may present as an increased susceptibility to infection, autoimmunity, autoinflammatory disease, allergy, bone marrow failure, and/or malignancy [1, 2]. Secondary immunodeficiency disease (SID; or acquired forms of immunodeficiency) is caused by external factors that include underlying diseases (such as cancer) or medication (including steroids) [3]. Patients with PIDs or SID experience increased morbidity and mortality owing to recurrent and severe infections that lead to a reduced quality of life [3, 4].

The current standard of care for patients with PIDs and an antibody deficiency is lifelong immunoglobulin replacement therapy (IgRT), delivered by intravenous infusion (IVIG) or subcutaneous infusion (SCIG) [5–7]. Standard IVIG therapy typically involves one infusion per month at a maintenance dose of 0.4–0.6 g/kg [8]. SCIG therapy is typically administered once weekly or every two weeks at a maintenance dose of 0.1 g/kg/week [8], with difference in infusion schedules largely dependent on patient preference. Although treatment with IVIG is effective in preventing infection, it can be associated with adverse events (AEs), including headaches, fever, allergies, and other systemic reactions [7]. Additionally, the requirement for repeated venous access and patient visits to physician offices or outpatient infusion centres may negatively affect patient health-related quality of life (HRQoL) [7], although this may vary by region. For example, in Canada, IVIG can only be administered in a healthcare setting by a healthcare professional (HCP) [9]; in comparison, IVIG may be administered at home independently in the UK or with nursing support in the USA [10, 11], both of which may lessen some of the patient burden of visiting a healthcare centre.

Importantly, SCIG offers an alternative therapy for patients who experience AEs using IVIG or have difficulty with venous access. SCIG infusion has been shown to be as effective as IVIG at preventing infections in patients with PIDs, and results in fewer systemic adverse reactions [7, 8, 12]. However, patients who report less satisfaction with SCIG than IVIG often identify increased frequency of infusions and local site reactions as primary reasons [8]. Although SCIG infusions require more frequent administration than IVIG, SCIG can be more easily administered at home, and patients/caregivers can adjust the method of delivery, infusion volume, infusion rate, number of sites, and number of infusions per week, depending on patient preference and needs [7, 12]. Additionally, estimates suggest that the annual cost of SCIG to healthcare systems is less than IVIG, owing to the lower direct costs associated with medical supplies and the reduced nursing support required for administration [13].

Several SCIG formulations are available for the treatment of patients with PIDs, including differing immunoglobulin (Ig) G (IgG) concentrations (10%, 16%, 16.5%, and 20%) [14–16] and infusion with recombinant human hyaluronidase (an enzyme that depolymerizes hyaluronan in the extracellular matrix to transiently increase tissue permeability to Ig) [7, 17]. SCIG delivery facilitated by recombinant human hyaluronidase enables the delivery of larger volumes of IgG at a single infusion site every 3–4 weeks and can be administered at the same dose as a patient’s previous IVIG therapy [7, 12]. The first liquid IgG 20% formulation approved globally for subcutaneous administration in patients with PIDs was immune globulin subcutaneous (human) 20% solution, stabilized with proline (IgPro20; Hizentra [CSL Behring, King of Prussia, PA, USA]) [18]. Compared with less-concentrated SCIG therapies, SCIG 20% allows for smaller infusion volumes and higher infusion rates [19]. Between April and September 2018, the Canadian Blood Services formulary phased in another SCIG 20% therapy: immune globulin subcutaneous (human) 20% solution, stabilized with glycine (Ig20Gly; Cuvitru [Baxalta US, Inc., a Takeda company, Lexington, MA, USA]) [20, 21].

In Europe, Ig20Gly is indicated as a replacement therapy in adults and children of any age for PIDs associated with impaired antibody production, and for SID in patients who experience severe or recurrent infections, ineffective antimicrobial treatment, and either proven specific antibody failure or a serum IgG level of less than 4 g/L [22]. For patients with PIDs, after steady-state IgG levels are attained, it is recommended that maintenance doses are administered at repeated intervals to reach a cumulative monthly dose of 0.3–1.0 g/kg [22]. In the USA, Ig20Gly is indicated as a replacement therapy for PIDs with antibody deficiency in adult and paediatric patients aged 2 years and above [23]; Ig20Gly can be administered at regular intervals, daily, or up to every 2 weeks, depending on the patient’s pharmacokinetic and clinical response profile [23]. Two pivotal phase 2/3 clinical trials conducted in North America (NCT01218438) and Europe (NCT01412385) demonstrated favourable efficacy, safety, and tolerability of Ig20Gly delivered by infusion pump in patients with PIDs; 4327 infusions were administered in 74 patients in the North American trial and 2349 infusions were administered in 49 patients in the European trial [19, 24]. Overall, both trials showed that Ig20Gly can establish protection against infection with stable steady-state IgG levels [19, 24].

Following the European approval in November 2021 of manual administration of IgPro20 via a syringe for use in patients with PIDs [25], manual administration of Ig20Gly via a syringe was approved in Europe in September 2023 as an alternative to infusion pump administration [22]. Manual administration of SCIG avoids technical and logistical requirements associated with infusion pump use (e.g. software problems, battery failures, or inadequate interface design), permitting easier infusion at home [26]. Importantly, prior studies using manual administration for the delivery of SCIG therapies have demonstrated the efficacy, safety, and tolerability of manual administration in adults and paediatric patients with PIDs; these studies are summarized in Table 1 [15, 16, 27–31].

**Table 1:** Studies of manual administration of SCIG therapies other than Ig20Gly

	Cowan et al. 2021 [27]	Patel et al. 2015 [28]	Shapiro 2010 [16]	Shapiro 2013a [30]	Shapiro 2013b [29]	Walter et al. 2020 [31]	Warnatz et al. 2022 [15]
Type of study, location	Phase 4, open-label multicentre study of high SCIG infusion rates in patients with PIDs in the USA	Retrospective chart review of paediatric patients with PIDs in North America	Retrospective chart review of patients with PIDs in the USA	Retrospective chart review of patients with PIDs in the USA	Retrospective chart review of patients with PIDs in the USA	Retrospective chart review of patients with PIDs enrolled in the SCIG manual administration program in Canada	Multicentre, open-label, randomized, non-inferiority trial in adults with PIDs in Australia, Germany, Italy, and the UK
Demographics	Adults and adolescents, n = 16	Paediatric patients, n = 88	Adults and paediatric patients, n = 104	Adults and paediatric patients, n = 173	Paediatric patients, n = 96	Adults, n = 62	Adults, n = 30
SCIG	SCIG 20% (IgPro20; Hizentra)	SCIG 20% (IgPro20; Hizentra)	SCIG 16% (Vivaglobin)	SCIG 16% (Vivaglobin) or SCIG 20% (IgPro20; Hizentra)	SCIG 16% (Vivaglobin) or SCIG 20% (IgPro20; Hizentra)	SCIG 16% (Vivaglobin), SCIG 20% (IgPro20; Hizentra) or SCIG 20% (Ig20Gly; Cuvitru)	SCIG 16.5% (Gammanorm)
Study objective	To evaluate the safety and tolerability of IgPro20 manual administration infusions at flow rates of 0.5–2.0 mL/min	To analyse the safety and efficacy of SCIG 20% in children aged < 5 years	To evaluate dosing and administration patterns, IgG trough levels, safety, and tolerability of manual administration compared with administration by infusion pump in patients who chose their SCIG administration method	To evaluate dosing and administration patterns, characterize serum IgG levels, and evaluate the safety and tolerability of the manual administration technique of patients receiving SCIG treatment	To analyse usage patterns, pharmacokinetic responses, and safety outcomes of paediatric patients using SCIG administered manually and by infusion pump	To determine the effectiveness of SCIG manual administration in adult patients with PIDs	To evaluate patient HRQoL and satisfaction with SCIG 16.5% when administered by infusion pump or manually
Inclusion criteria	Patients with PIDs receiving a stable dose of IgPro20 therapy at a flow rate of ~ 0.5 mL/min/site for ≥ 1 month prior to the first day of the study	Patients with PIDs who had received > 1 dose of SCIG 20% prior to 5 years of age, regardless of previous Ig replacement therapy	Patients who were either Ig-naive or were switched from IVIG to a 16% SCIG; patients who had received ≥ 1 course of SCIG therapy between 1 January 2006 and 1 April 2008 for PIDs	Patients of any age who had received ≥ 1 course of SCIG therapy for PIDs between 1 January 2006 and 1 November 2011	Patients of any age who received ≥ 1 course of SCIG therapy for PIDs between 1 January 2006 and 1 November 2011	Patients aged ≥ 18 years with PIDs who had received SCIG via manual administration for ≥ 12 consecutive months	Patients aged ≥ 18 years with PIDs who had received SCIG for ≥ 1 month
Key results	Subcutaneous IgPro20 manual administration infusions at flow rates up to 2.0 mL/min were well tolerated Rates of treatment-related local TEAEs were 0.023, 0.082, and 0.025 per infusion for 0.5, 1.0, and 2.0 mL/min flow rates, respectively Median volume administered was 55.0 mL and median dose was 127.3 mg/kg	Manual administration of SCIG 20% was tolerated without difficulty and permitted faster administration times in some patients than administration via infusion pump	Overall, 65/74 patients (88%) who initiated SCIG therapy using manual administration remained on manual administration and 13/29 (45%) who started on infusion pump switched to manual administration Mean serum IgG levels were comparable between patients using manual administration (12.3 g/L) and infusion pump (11.5 g/L) AEs were reported in 31% of patients. Local infusion site reactions occurred in 28% of patients using manual administration and 28% of patients using an infusion pump	Overall, AEs were reported once every 5–6 visits and were less frequent with manual administration than infusion pump Approximately two-thirds of patients chose to initiate SCIG therapy with manual administration and most continued to use manual administration Manual administration resulted in acceptable serum IgG levels and allowed for faster administration than infusion pump	Manual administration had a favourable safety profile and was a viable alternative to infusion pumps in children Serum IgG levels were 17.8% higher with subcutaneous manual administration than with infusion pump The frequency of AEs was generally lower among patients using manual administration than infusion pump (87% lower in patients aged < 2 years and 50% lower in patients aged 2 to < 10 years) The majority of AEs for both infusion pump and manual administration were local and self-limiting Manual administration allowed for faster infusion times (most completed in < 10 min) than infusion pump	Manual administration of SCIG was reported to result in adequate steady-state IgG levels Only 8 patients discontinued SCIG following ≥ 12 months of treatment via manual administration	Compared with infusion pumps, for manual administration the number of infusions per site was > 2 times higher, total infusion rate was > 3 times higher, and total time expenditure for dosing was shorter The 3-month rate of infection and residual serum Ig levels were comparable for infusion pump and manual administration HRQoL did not significantly differ between patients using manual administration and those using administration via infusion pump Direct treatment costs, excluding cost for SCIG 16.5%, were lower for manual administration than for infusion pump; indirect costs were similar for both modes of administration

*AE* adverse event, *HRQoL* health-related quality of life, *Ig* immunoglobulin, *IgG* immunoglobulin G, *IgPro20* immune globulin subcutaneous (human) 20% solution, stabilized with proline, *Ig20Gly* immune globulin subcutaneous (human) 20% solution, stabilized with glycine, *PID* primary immunodeficiency disease, *SCIG* subcutaneous immunoglobulin, *SD* standard deviation, *TEAE* treatment-emergent adverse event

During the COVID-19 pandemic, owing to patient preference for at-home treatment and a reduced availability of infusion pumps, there was accelerated uptake of SCIG manual administration. Nonetheless, practical guidance and assessment of patient suitability for manual administration remains limited. Therefore, this article reviews infusion parameters, safety, patient-reported outcomes (PROs), and the economic benefits of Ig20Gly manual administration, as well as providing practical guidance for use of this method.

### Studies reporting outcomes following the manual administration of Ig20Gly

To date, two published studies have discussed infusion parameters, safety, and PROs following treatment with Ig20Gly infused via manual administration. The first was the CANadian CUvitru Non-interventional study (CANCUN; NCT03716700) of patients with PIDs or SID transitioning to Ig20Gly from a prior SCIG therapy; this publication included a subgroup analysis in which manual administration of Ig20Gly was evaluated [20]. CANCUN was a phase 4, prospective, single-arm study in six centres across Canada (excluding Quebec) with a maximum 12-month (− 1/ + 2 months) follow-up period [20]. Overall, 125 patients aged 2 years and above with PIDs or SID were included; of these, 54 patients (43.2%) infused Ig20Gly via manual administration [20]. Of patients infusing by manual administration, median (range) age was 63 (19–82) years and 72.2% of patients were female; in total, 51.9% of patients infusing manually had PIDs and 48.1% had SID [20]. The second publication reported on a retrospective analysis of the IG-TATRY (NCT04636502) study, which was conducted to analyse real-world data on the use of Ig20Gly in paediatric patients with PIDs from four immunology/haematology clinics in Poland [32]. In total, 75 paediatric patients (aged < 18 years) with PIDs were included, of whom 16 (21.3%) infused Ig20Gly by manual administration (7 patients [9.3%] infused only by manual administration; 9 patients [12.0%] used both infusion pump and manual administration). Of patients using manual administration, 3 patients were aged 6 years and under, 7 patients were aged 7–11 years, and 6 patients were aged 12–17 years [32].

#### Infusion parameters of manually administered Ig20Gly

In the CANCUN study, compared with patients using an infusion pump at the 12-month follow-up, patients using manual administration did so with a lower median (interquartile range [IQR]) volume per infusion (30.0 [20.0–40.0] mL vs 43.0 [40.0–60.0] mL) [20]. The median (IQR) volume per site infused by manual administration was lower in the IG-TATRY study (12.5 [10.0–20.0] mL) than in the CANCUN study, likely owing to the enrolment of only paediatric patients in the IG-TATRY study (Table 2) [32]. Consistent with age-dependent infusion volumes, median volume per site was shown to increase with age in the IG-TATRY study, starting at 10.0 mL in patients aged up to 6 years, 15.0 mL in patients aged 7–11 years, and 17.5 mL in patients aged 12–17 years [32]. However, despite the difference in reported volumes per infusion between infusion pump and manual administration, it is possible to administer similar volumes of Ig20Gly using either method. For the first infusion by manual administration, 20.0 mL per site is generally considered appropriate to assess patient tolerability. The typical maximum syringe volume used for manual administration is 30.0 mL, but patients can infuse greater volumes, if tolerated, by switching the type of syringe.

**Table 2:** Infusion parameters of Ig20Gly manual administration

Infusion parameter	CANCUN study [20]	IG-TATRY study [32]
Infusion volume, mL, median (IQR)	30 (20–40)^a^	13 (10–20)^b^
Infusion duration, min, median (IQR)	24 (10–40)	NR
Dose received, g/kg, monthly, median (IQR)	0.4 (NR)^c^	0.3 (0.2–0.4)
Number of infusions/month, median (IQR)	4 (4–8)	3.5 (3.0–4.0)
Dosing interval, n (%)		
Daily 2–6 times/week Once weekly Bi-weekly Other	0 17 (40.5) 25 (59.5) 0 0	NR NR NR NR NR

*CANCUN* CANadian CUvitru Non-interventional, *Ig20Gly* immune globulin subcutaneous (human) 20% solution, stabilized with glycine, *IQR* interquartile range, *NR* not reported

^a^Per infusion

^b^Per site

^c^Median monthly dose by bodyweight was reported for the whole cohort, regardless of method of administration (infusion pump or manual administration); median (IQR) weekly dose for the manual administration cohort was 8.0 (6.0–8.0) g

With respect to infusion duration, in the CANCUN study at 12 months, the median (IQR) duration of infusion was shorter for patients using manual administration than for use of an infusion pump (24 [10–40] min vs 60 [45–73] min) [20]. Consistent with these observations, reduced infusion time with manual versus infusion pump administration was demonstrated in a retrospective chart review of 173 paediatric and adult patients with PIDs receiving IgPro20 (< 9 min vs 49 min) [30]. Two other studies reported similar infusion times for manual administration of IgPro20, ranging from a mean duration of 23 min to 47 min [27, 28]. One potential modifier of infusion duration is the rate of infusion, with one study showing a mean weekly duration of IgPro20 administered by manual administration of 23–28 min at a 2.0 mL/min infusion rate and 103–108 min at a 0.5 mL/min infusion rate [27].

Overall, in the CANCUN study at 12 months, median (IQR) infusion rate for manual administration was 30.0 (30.0–60.0) mL/h/site (unpublished observation), compared with 40.0 (34.0–59.0) mL/h/site for all patients [20]. By comparison, for the paediatric population in the IG-TATRY study, patients using manual administration had a higher median (IQR) infusion rate than those using an infusion pump (92.5 [50.0–170.2] mL/h and 40.0 [29.5–55.5] mL/h, respectively; unpublished observations). Notably, the higher infusion rates in the IG-TATRY study may result from differences in age (paediatric vs adult) or rate calculations (mL/h vs mL/h/site); rate per infusion site was not reported in the IG-TATRY study.

In the IG-TATRY study, paediatric patients using manual administration received a lower median (IQR) monthly dose of 0.3 (0.2–0.4) g/kg than the entire cohort (0.4 g/kg), which also included patients who used an infusion pump [32]. The higher reported median monthly dose of Ig20Gly for the entire cohort was similar to doses reported in prior studies: 0.40–0.55 g/kg between 6- and 12-month follow-up visits [33, 34]. Additionally, for the 16 patients using manual administration in the IG-TATRY study, the median (IQR) infusion interval was 8.5 (7.0–10.0) days, equating to a median (IQR) of 3.5 (3.0–4.0) infusions per month [32].

In the CANCUN study, patients using manual administration were more likely to use two or fewer infusion sites than patients using an infusion pump (96.3% vs 55.0%) [20]; more patients infused at three or more sites using an infusion pump than using manual administration (45.0% vs 3.7%). Studies with other SCIG therapies (including IgPro20 and SCIG 16% [Vivaglobin; CSL Behring GmbH, Marburg, Germany]) were concordant with these findings, showing that patients using manual administration typically infuse at two or fewer sites [16, 28]. Patients administering SCIG 16% using an infusion pump were more likely to infuse at three or more sites than patients using manual administration [16]. Individual patient dexterity, leading to difficulty infusing at more than two infusion sites for any given infusion via manual administration, may contribute to this observation of increased infusion frequency to achieve the target dose. However, with the support of a caregiver, it is possible to use multiple syringes for manual administration at more than two sites simultaneously.

Patients infusing by manual administration in the CANCUN study also tended to infuse at more frequent dosing intervals than those using an infusion pump (40.5% of patients infused 2–6 times/week for manual administration, compared with 3.1% of patients infusing at this frequency via an infusion pump) [20]. Nevertheless, for both manual administration and infusion pump administration, the most frequent dosing interval was once weekly (59.5% and 87.5% of patients, respectively) [20].

#### Efficacy, safety, and tolerability of Ig20Gly infusion by manual administration

Median (IQR) serum IgG trough levels in the CANCUN study were similar for manual administration (9.1 [8.3–11.0] g/L) and infusion pump cohorts (8.6 [7.9–10.6] g/L) [20]. Consistent with this observation, serum IgG trough levels in the IG-TATRY study were comparable between paediatric patients who infused Ig20Gly by manual administration and the overall population (patients who infused with manual administration or infusion pump) [32]. In the IG-TATRY study, the median (IQR) serum IgG trough level was 9.0 (8.2–9.6) g/L for patients using manual administration (measured 1–7 days post-dose) and 8.0 (7.0–9.3) g/L for the entire cohort (measured 1–14 days post-dose; it was not possible to analyse the serum IgG trough levels up to 14 days post-dose in patients using manual administration because of an insufficient number of IgG measurements) [32]. Importantly, in all patients in both studies, the median IgG levels were above a putative minimum protective threshold of 5.0 g/L in all age groups (although in clinical practice, higher thresholds are sometimes used depending on individual patient characteristics) [22, 35–37]. Considering delivery of any SCIG therapy, serum IgG trough levels following manual administration are often higher than those following infusion pump administration [16, 29, 30, 38, 39], although no pharmacokinetic analyses have been conducted to explain these observations.

In the IG-TATRY study, Ig20Gly was well tolerated. Overall, five patients (6.7%) discontinued the study; no patient reported manual administration of Ig20Gly as the reason for study discontinuation [32]. Reasons for study discontinuation included no requirement for further treatment owing to satisfactory and stable IgG levels, patient request, and a single death in an 8-year-old patient with PID (no AEs were reported for this patient during the study period) [32]. Tolerability findings were not explicitly reported for patients using manual administration in the CANCUN study.

In the CANCUN study, for patients using infusion pump and manual administration, the number of reported AEs of interest (defined as any AE described as a warning/precaution in the product monograph, reported in a previous trial, or observed during post-marketing surveillance) was similar: 9 AEs of interest in 6/71 (8.5%) patients and 14 AEs of interest in 10/54 (18.5%) patients, respectively [20, 40]. All AEs of interest associated with manual administration were mild or moderate in nature (n = 7 each in 7 and 4 patients, respectively); for patients using an infusion pump, 7 AEs were reported in 4 patients as mild, 1 AE was reported in 1 patient as moderate, and 1 AE was reported in 1 patient as severe (defined as any event that interrupted usual activity of daily living, significantly affected clinical status, or may require therapeutic intervention) [20]. Of the AEs of interest associated with manual administration, 5 were considered related, 5 were possibly related, and 1 was probably related to Ig20Gly infusion; no serious AEs were reported in patients using manual administration [20]. The reported safety profiles associated with manual administration of Ig20Gly were consistent with studies using other SCIG therapies [16, 27]. For example, in one study of IgPro20 delivered by manual administration, most AEs were local and mild and tended to subside over time [16]. However, the IG-TATRY study did not report safety outcomes because the study objective was to report real-world data on treatment regimens, patient characteristics, and clinical outcomes from Ig20Gly treatment [32]. As such, additional studies in paediatric patients may prove beneficial in validating safety outcomes in this population.

#### Patient-reported HRQoL outcomes following manual administration of Ig20Gly

**Fig. 1:**
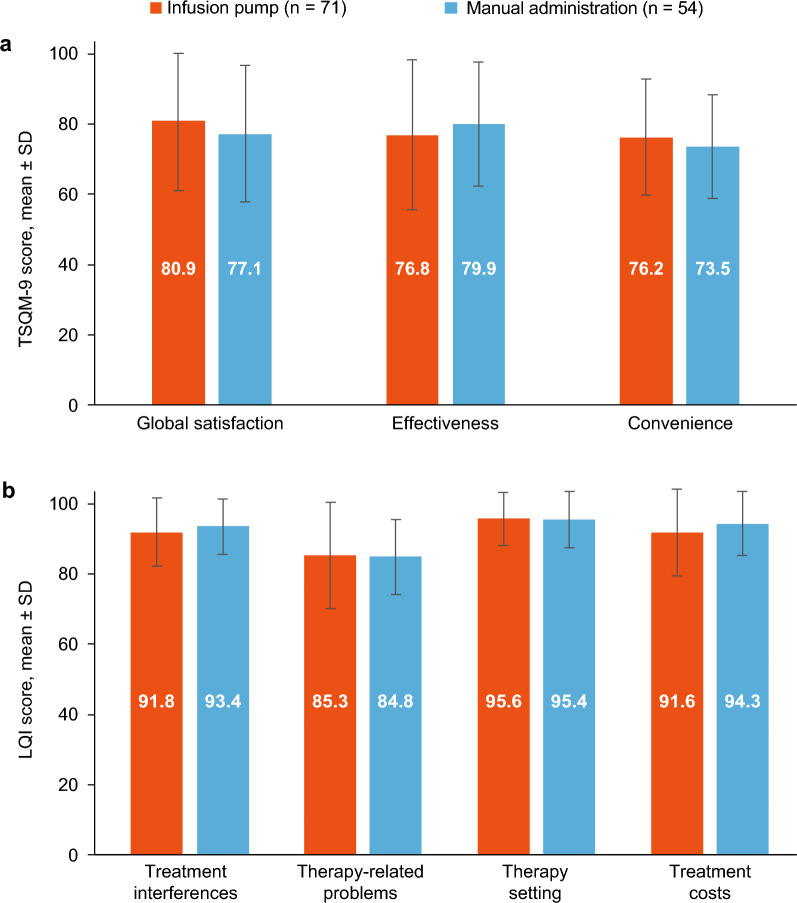
TSQM-9 (**a**) and LQI (**b**) scores following manual administration of Ig20Gly in the CANCUN study. Values presented for patients who provided feedback; the number of patients with available data was not provided for each subcategory of the TSQM-9 or LQI scores. The TSQM-9 recall period was 2–3 weeks from the study visit date or since the last Ig20Gly administration. For TSQM-9 and LQI, scores for each domain were calculated by summing the items in each domain and then transforming into a value ranging from 0 to 100 (higher scores indicated better satisfaction/status). *CANCUN* CANadian CUvitru Non-interventional, *Ig20Gly* immune globulin subcutaneous (human) 20% solution, stabilized with glycine, *LQI* Life Quality Index, *SD* standard deviation, *TSQM-9* 9-item Treatment Satisfaction Questionnaire for Medication

The choice of manual or infusion pump administration may be influenced by availability of at-home support, patient dexterity and strength, confidence in infusing using manual administration, adherence, and an ability to fit manual administration around daily schedules. In the CANCUN study, PROs, as measured by the 9-item Treatment Satisfaction Questionnaire for Medication scores (including global satisfaction, effectiveness, and convenience), Life Quality Index (including treatment interferences, therapy-related problems, therapy setting, and treatment costs), and Treatment Preference Questionnaire data were similar between patients who administered Ig20Gly manually or by infusion pump (Fig. 1) [20]. After 12 months, all patients, regardless of mode of administration, expressed an interest in continuing Ig20Gly treatment [20]. These data are in accordance with studies of other SCIG therapies, which identified comparable levels of patient satisfaction with manual and infusion pump administration [15], suggesting that patient suitability for manual administration is in part dependent on patient preference. HCPs should encourage patients to try both methods of administration and the option used may be changed over time to suit the need of the patient/caregiver according to preference or personal circumstances.

### Patient suitability for manual administration of SCIG

Body mass index (BMI) has been investigated to determine if it is a factor that may alter patient success with manual administration. One study of patients with PIDs receiving IgPro20 to assess the safety and tolerability of increasing manually administered infusion flow rates (from 0.5 mL/min to 2.0 mL/min) included a responder analysis (patients who completed a pre-defined minimum number of infusions) with stratification by patient BMI [27]. At all flow rates considered, there were no meaningful differences in the proportion of responders in a population of obese (BMI ≥ 30 kg/m^2^) and non-obese (BMI < 30 kg/m^2^) patients. At the 0.5 and 1.0 mL/min infusion rates, there was a 100% responder rate; at the 2.0 mL/min infusion rate, there was an 87.5% responder rate [27]. However, no underweight patients (BMI ≤ 18 kg/m^2^) were enrolled into the study and thus conclusions could not be drawn on the safety and tolerability of manual administration in this group [27].

Additionally, a retrospective chart review of 40 patients who were obese with PIDs using manual administration of SCIG 16% (Vivaglobin) identified differences between manual administration and infusion pump administration in obese patients [38]. Mean serum IgG levels in obese patients were higher using manual versus infusion pump administration (10.0 g/L and 8.4 g/L, respectively) [38]. For patients using manual administration, the mean (SD) monthly SCIG dose was lower in obese patients than non-obese patients (0.5 [0.2] g/kg vs 0.6 [0.2] g/kg), yet mean (SD) weekly SCIG volume was higher in obese patients than non-obese patients (72.1 [31.7] mL vs 46.2 [24.7] mL) [38]. Furthermore, obese patients using manual administration infused more times per week than non-obese patients (mean 3.3 days vs 2.7 days) but used a similar number of sites per infusion (1.5 sites vs 1.3 sites, respectively). For all patients, AE rates were lower in patients using manual administration than infusion pump administration (15.6% vs 20.7% of visits, respectively); rates of overall and local AEs were also slightly lower in obese than non-obese patients (15.8% vs 17.6% of visits, respectively) [38].

Despite these results, in clinical practice BMI is not considered to be a good indicator of patient success with manual administration. Instead, patient strength, support, and confidence with use of a syringe appear to be key determinators of patient suitability for manual administration. However, further studies are warranted to confirm any differences in patient suitability based on BMI for Ig20Gly delivery by manual administration.

### Economic impact of SCIG manual administration

Manual administration of SCIG is expected to be more economical than pump-assisted infusion of SCIG or IVIG owing to fewer supplies and a reduced requirement for nursing support. A Canadian economic simulation model suggested that replacing IVIG delivered by infusion pump with manually administered SCIG in 50% of adult patients with PIDs would result in a cost saving for the healthcare system of CAN$5736 per patient within the first 3 years of therapy, which would represent overall reduced costs of CAN$1.3 million for the population of patients with PIDs in British Columbia [13]. Additionally, the economic model suggested that switching from IVIG to a pump-based SCIG option would result in cost savings of CAN$1621 per patient and CAN$369 665 for the population of patients with PIDs in British Columbia over the first 3 years of therapy [13]. Additionally, an earlier clinical trial in adults with PIDs in Europe also identified lower monthly direct costs (including expenses for Ig, pumps/injection kits, and nursing time) associated with manual administration than infusion pump administration (mean ± SD [range]: €100.2 ± 65.8 [22.5–283.5] and €178.2 ± 102.6 [64.4–464.6], respectively) [15].

### Practical guidance on the manual administration of Ig20Gly

For infusion of Ig20Gly by manual administration, a syringe and butterfly needle are used. Patients should gradually push down on the plunger of the filled syringe (as directed by their HCP) until all fluid in the syringe has been injected [41]. For patients with dexterity issues, syringe holders (for example, SteadyJect [CSL Behring, King of Prussia, PA, USA]) may be used to facilitate infusions. Availability of pre-filled syringes may reduce errors in the preparation of infusion equipment [42]. Initially, it is recommended that patients start with an infusion speed of 1.0–2.0 mL/min to prevent discomfort [41]; however, the maximum infusion rate via manual administration has not been well defined and should be adjusted in line with patient tolerability. Notably, the US prescribing information for Ig20Gly states a maximum infusion rate of 60 mL/h/site for administration of Ig20Gly by infusion pump while the EU summary of product characteristics is open-ended, noting that the infusion rate may be increased as tolerated by the patient [22, 23]. However, patient tolerability of treatment is highly variable; if there are concerns over tolerability, Ig20Gly may be delivered at full target dose but at a reduced rate. Some HCPs may also recommend starting treatment using a dose ramp-up schedule regardless of prior experience with SCIG, and thus an individualized approach should be taken.

Ig20Gly can be administered using a syringe at a single infusion site; if additional infusion sites are required, a new sterile syringe should be used or a bifurcated needle set may be considered for multiple infusions [22, 23]. In paediatric patients, the infusion site can be changed every 5.0–15.0 mL; in adults, doses greater than 30.0 mL can be divided according to patient preference [22]. Ig20Gly dose adjustments may be considered on an individual basis depending on the patient’s initial trough serum IgG level achieved after the first infusion and/or recurrence of infection while the patient is receiving treatment. Indeed, one meta-analysis of 11 studies assessing IgG trough levels associated with SCIG and IVIG showed that for every 1 g/L increase in serum trough IgG level, there was a trend towards decreasing incidence of infection in patients receiving SCIG [43]. Additionally, the Canadian Prairie guidelines for the use of Ig state that for PIDs, the maintenance dose should be adjusted to achieve serum IgG trough levels of at least the lower limit of the age-specific serum IgG reference range, or as needed to achieve clinical effectiveness [44].

It is important to note that patients may be hesitant to use needles; therefore, face-to-face training by an infusion nurse may prove beneficial in building patient confidence to infuse by manual administration and also permits patients to ask more questions about their therapy. Information brochures, such as those developed by the International Patient Organisation for Primary Immunodeficiencies (https://ipopi.org/), may also aid patient confidence in disease management and treatment. However, there is a lack of formal training for nurses in the UK and wider Europe with regard to Ig infusion; instead, nurses who are experienced with subcutaneous infusions have acquired knowledge over their careers. Newly qualified nurses in Europe are calling for formal accreditation, similar to the Immunoglobulin National Society (IgNS) certification in the USA (https://ig-ns.org/ig-certification/), for performing or assisting with infusions to ensure that high-quality standardized training is available across countries.

## Conclusions

Manual administration of Ig20Gly has been shown to permit faster rates of infusion than administration via infusion pump. For manual administration of Ig20Gly, patients typically infused at two or fewer infusion sites; owing to patient dexterity, it may be challenging to infuse at multiple sites, which may increase infusion frequency with manual administration. However, at-home support from family, friends, or a primary caregiver may facilitate infusions at multiple sites at the same time. Manual administration of Ig20Gly has been reported to have a favourable safety profile and offers an effective and well-tolerated alternative to pump administration. Compared with administration via infusion pump, Ig20Gly manual administration resulted in a similar number and severity of AEs. PROs suggested comparable levels of satisfaction with manual or infusion pump administration of Ig20Gly, with patient preference being a key determinator of patient success with either method of administration. Economic studies estimate that manual administration of SCIG is more cost-effective than IVIG or pump-administered SCIG, resulting in potentially large savings for healthcare systems. Generally, for infusion of SCIG by manual administration, patients are advised to initiate administration at the full target dose at an infusion rate of 1–2 mL/min to minimize discomfort; however, the rate of infusion should be adjusted depending on patient tolerability. HCPs should assess individual patient serum trough IgG levels and rate of infections to assess the need for Ig20Gly dose adjustments after the initial infusion and throughout treatment. Face-to-face patient training on the use of a needle and syringe for Ig20Gly delivery by a nurse experienced with subcutaneous infusions may increase patient confidence in the use of manual administration of Ig20Gly.

## Data Availability

No data sets were generated or analysed during the current study.

## Abbreviations

AE: Adverse event
BMI: Body mass index
CANCUN: CANadian CUvitru Non-interventional
fSCIG: Subcutaneous immunoglobulin delivery facilitated by recombinant human hyaluronidase
HCP: Healthcare professional
HRQoL: Health-related quality of life
Ig: Immunoglobulin
IgG: Immunoglobulin G
IgPro20: Immune globulin subcutaneous (human) 20% solution, stabilized with proline
IgRT: Immunoglobulin replacement therapy
Ig20Gly: Immune globulin subcutaneous (human) 20% solution, stabilized with glycine
IQR: Interquartile range
IVIG: Intravenous immunoglobulin
LQI: Life Quality Index
NR: Not reported
PID: Primary immunodeficiency disease
PK: Pharmacokinetic
PRO: Patient-reported outcome
SC: Subcutaneous
SCIG: Subcutaneous immunoglobulin
SD: Standard deviation
SID: Secondary immunodeficiency disease
TEAE: Treatment-emergent adverse event
TSQM-9: 9-Item Treatment Satisfaction Questionnaire for Medication
